# Improving risk prediction in hypertrophic cardiomyopathy: the key role of Dutch founder variants

**DOI:** 10.1007/s12471-021-01581-8

**Published:** 2021-05-10

**Authors:** R. Walsh, Y. M. Pinto

**Affiliations:** Department of Clinical and Experimental Cardiology, Amsterdam Cardiovascular Sciences, Amsterdam University Medical Centers, location Academic Medical Center, University of Amsterdam, Heart Center, Amsterdam, The Netherlands

Hypertrophic cardiomyopathy (HCM) is an important cardiac condition that can lead to sudden cardiac death (SCD) or heart failure in a subset of patients. Although HCM has been the subject of intense investigation for several decades now, our ability to predict disease onset and progression—in particular, to identify those patients at highest risk of severe clinical events—remains limited and represents one of the key challenges facing clinicians and researchers in this field. Genetic testing is a useful but blunt tool for risk prediction in HCM patients and their families. Causative rare variants in the index patient (identified in 30–50% of cases) enable cascade screening and the discharge from ongoing clinical surveillance of relatives without the variant.

However, the factors that influence disease penetrance (which pathogenic variant carriers will develop the phenotype?) remain poorly understood, and asymptomatic genotype-positive individuals require regular and costly cardiac screening to monitor disease onset. While some risk prediction models are recommended for HCM patients, e.g. the HCM Risk-SCD calculator for prioritising patients who may benefit from prophylactic implantable cardioverter defibrillators [1], more comprehensive and accurate predictors of clinical severity are required.

The BIO FOr CARe (Identification of BIOmarkers of hypertrophic cardiomyopathy development and progression in Dutch *MYBPC3* FOunder variant CARriers) cohort described in this issue of the *Netherlands Heart Journal *[2], is an important initiative designed to tackle this issue. While single-centre studies have provided some insights into genotype-phenotype correlation in HCM and the factors associated with clinical progression [3], the genetic and clinical heterogeneity of HCM limit the insights that can be achieved by this approach.

Recently, large patient registries have demonstrated the benefits of multicentre collaborative approaches to explore the complex aetiologies underlying relatively rare diseases such as HCM. The Sarcomeric Human Cardiomyopathy Registry (https://theshareregistry.org) [4] includes data from 16 research centres and has enabled large-scale studies, including those investigating the association between age of diagnosis, genotype and disease outcomes [5]. The HCM Registry (https://hcmregistry.org) involves 40 research sites and has recruited 2750 patients to explore the relationship between genotype and cardiac-imaging metrics [6] and the contribution of common genetic variation to disease risk [7].

While the scale of these registries offers considerable advantages, efforts to investigate the factors underlying clinical variability in HCM are hindered by the substantial genetic heterogeneity in these cohorts. Several hundred distinct pathogenic variants in sarcomere genes are observed in the registry cohorts, while at least half of the cases are genotype-negative and are expected to have a more complex, non-Mendelian aetiology.

In contrast, BIO FOr CARe takes advantage of the unique genetic architecture of HCM in the Netherlands: four truncating variants in *MYBPC3* (three of which are founder variants) account for at least half of all Dutch HCM probands where a causative variant is found [8]. These variants are expected to act through a common disease mechanism (haploinsufficiency of cardiac myosin-binding protein C) and therefore allow for the investigation of disease modifiers in the absence of confounding effects from the primary genetic variants. The prominence of these *MYBPC3* variants in Dutch HCM patients also facilitates the recruitment of currently phenotype-negative carriers into this cohort, through genetic screening of family members of HCM index cases (which is widely performed in the Netherlands) and potentially more widely through future genome-sequencing efforts in the general population. The inclusion of this broader range of phenotypes, from asymptomatic individuals to patients with one or more of the defined disease endpoints, is expected to empower the search for relevant biomarkers and disease modifiers.

In addition to these genetic factors, the Netherlands offers more advantages for developing such cohorts, including long established cardiac genetics programmes at the major university medical centres and a strong collaborative culture between these institutions. The longitudinal study design of this project is particularly advantageous, with blood collected every 2 years to monitor changes in biomarker concentrations and their association with phenotype progression. This will certainly require a substantial ongoing investment in resources and infrastructure, with findings from these aspects of the study expected to take several years to come to fruition.

Currently, the BIO FOr CARe biomarker projects focus on metabolomics by testing the hypothesis that acylcarnitine concentration is a marker of perturbed energy metabolism in HCM and by biomarker discovery through untargeted metabolomics studies. The role of common genetic variants in modifying disease severity will also be explored. The potential of this approach was recently demonstrated by the association of a HCM-derived polygenic risk score with variability in both left ventricular wall thickness and clinical events in Dutch carriers of pathogenic sarcomere gene variants [9].

Future challenges in this effort will involve completing recruitment (statistical analysis has highlighted a target of 1000 individuals) and validating findings in broader and more genetically diverse HCM populations. While BIO FOr CARe plans to include other HCM genotypes, validation in other HCM registries will be critical for applying new predictive approaches in more heterogeneous populations. Ideally, this will also be applied to genotype-negative HCM families, for which methods for assessing risk are currently limited, although the prognosis is generally more benign and familial aggregation is less common. Enhanced risk prediction for HCM patients and their relatives enabled by registries such as BIO FOr CARe offers the opportunity for improved personalised medicine and more targeted clinical intervention in the near future.
